# Temporary Storage of the Human Nasal Tissue and Cell Sheet for Wound Repair

**DOI:** 10.3389/fbioe.2021.687946

**Published:** 2021-07-21

**Authors:** Yoshiyuki Kasai, Tsunetaro Morino, Izumi Dobashi, Eri Mori, Kazuhisa Yamamoto, Hiromi Kojima

**Affiliations:** Department of Otorhinolaryngology, The Jikei University School of Medicine, Tokyo, Japan

**Keywords:** human nasal tissue, cell sheet, preservation, cryopreservation, ready-to-use, wound healing

## Abstract

Temporary storage of nasal tissues and nasal cell sheets, which entails transportation between hospitals and cell culture facilities, is an important issue in regenerative medicine. Herein, we investigated the preservation of chilled and frozen nasal tissues and expiry dates of ready-to-use nasal cell sheets. Although the cell number in preserved tissues was lower than that in fresh tissue, nasal cell sheets could be fabricated from tissues that had been refrigerated for 5 days and frozen–thawed over 5 days. Moreover, the nasal mucosal cell sheets were preserved in a non-hazardous buffer. The cell number, viability, and structure were not maintained in saline containing E-cadherin for 2 days; however, these were maintained in Hank’s balanced salt solution for 2 days, but not for 5 days. To assess the proliferation capacity of cells in the stored cell sheets, we performed cell sheet grafting assays *in vitro*. Cell sheets stored in Hank’s balanced salt solution for 2 days adhered to collagen gel and expanded normally. Our results show that nasal tissues can be stored temporarily in refrigerators or deep freezers, and Hank’s balanced salt solution can be used for preservation of ready-to-use cell sheets for a few days.

## Introduction

Intractable otitis media, cholesteatoma, and adhesive otitis media were successfully treated using autologous nasal mucosal cell sheet as a regenerative medicine in a clinical study (Yamamoto et al., 2017). To develop new regenerative medicines using autologous somatic cells, the expiration date of the final product should be defined (Hayakawa et al., 2015; Knoepfler, 2015; Lopez-Beas et al., 2020). We previously showed that both nasal tissue and nasal mucosal cell sheets can be transported for 3 h without a decrease in quality (Kasai et al., 2019). However, the expiration dates for both nasal tissue and its cell sheet are unknown. In a study on preservation of the lung tissue, considerable apoptosis of cells stored for 5 days at 4°C was observed (Abe et al., 2006), indicating that the expiration date for the lung tissue preserved in refrigerator could be 5 days. Moreover, cells from cryopreserved umbilical cord or adipose tissues have the ability to grow and differentiate (Shimazu et al., 2015; Arutyunyan et al., 2018; Zanata et al., 2018); not only suspended cells, native tissues can also be stored in a deep freezer. In this context, we hypothesized that the nasal mucosal tissue could be preserved in refrigerator for 5 days and can be potentially preserved in deep freezer. Because the medium used for preservation of the cell sheet (the final product) contains some adventitious agents, it must be completely washed off. Hank’s balanced salt solution (HBSS)-based buffers reportedly keep the morphology of oral keratinocyte sheet intact for 7 days (Katori et al., 2016). We hypothesized that the nasal mucosal cell sheet could be preserved in HBSS for at least a few days.

In this study, we evaluated the expiration date of nasal mucosal tissue in keratinocyte culture medium (KCM), in which nasal tissue can be preserved for at least 3 h without contamination, as well as the expiration date of nasal cell sheets in non-hazardous buffer, namely HBSS and normal saline. We analyzed the cell number, viability, and the proliferative capacity of nasal mucosal cells in the cell sheets using previously established *in vitro* assays (Kasai et al., 2020) to compare various parameters before and after preservation.

## Methods

### Preparation and Preservation of Nasal Mucosal Tissue

All the experiments were performed in accordance with the Declaration of Helsinki and Ethical Guidelines for Medical and Health Research Involving Human Subjects in Japan. This study was approved by the Institutional Review Board of the Jikei University, Tokyo, Japan. All 13 patients, who were scheduled to undergo endoscopic sinus surgery, provided informed consent. Nasal mucosal tissue was collected from inferior nasal turbinate mucosa during endoscopic sinus surgery. The collected nasal mucosal tissue was disinfected using povidone-iodine. Depending on the experiment, tissues were maintained in KCM at 4°C or in freezing medium at −80°C (Stem-Cellbanker, GMP-grade, Nippon Zenyaku Kogyo, Fukushima, Japan). None of the volunteers was infected with human immunodeficiency virus, syphilis, or hepatitis B and C virus.

### Cell Expansion

KCM containing 10 μM Rho-associated kinase inhibitor (Y-27632, Wako Pure Chemical) was prepared, and explant culture was performed, as previously described by us (Kasai et al., 2020). Briefly, washed nasal mucosal tissue samples were cut into cubes (1.5 mm^3^); 32 cubes were placed on eight cell culture dishes (60 mm Primaria dish, Corning, Inc., Corning, NY, United States) and incubated at 37°C in an atmosphere of 5% CO_2_. Following 13 days of culture, the cells were collected by treatment with trypsin EDTA. In subculture, 3T3-J2 cells irradiated with X-ray from a J-TEC (Aichi, Japan) were seeded at a density of 4.0 × 10^4^ cells/cm^2^ as the feeder layer. Epithelial cells were seeded at a density of 1.0–3.0 × 10^4^ cells/cm^2^ on the 3T3-J2 cells. Following 4–7 days of culture, the cells were collected and stored in freezing medium.

### Cell Sheet Culture and Preservation

The 3T3-J2 cells were seeded into temperature-responsive cell culture substrate (CellSeed, Tokyo, Japan) in KCM with 10 μM Y-27632 and incubated for over 2 h. Expanded nasal mucosal epithelial cells were thawed and seeded at a density of 3.0 × 10^4^ cells/cm^2^ over the 3T3-J2 cell layer. The medium in the dishes was replaced with KCM on days 3, 5, and 7. The cells were detached as a cell sheet on day 8. For the preservation test, confluent cells were submerged in HBSS or saline.

### Histological Analysis

Histological analysis was performed as described in our previous article (Kasai et al., 2019). All sections were fixed in 4% paraformaldehyde (Wako Pure Chemical), embedded in paraffin, and sliced into 4 μm-thick sections. The sections were deparaffinized and stained with hematoxylin and eosin (HE; both from Wako Pure Chemical Industries, Osaka, Japan). For immunohistology, antigens were activated by autoclaving (121°C, 10 min) with citrate buffer (Histo-VT One, Nacalai Tesque, Kyoto, Japan). Non-specific reactions were prevented by peroxidase blocking (Dako, Carpinteria, CA, United States) and protein blocking (Nacalai Tesque). The sections were incubated overnight with the primary antibodies, namely anti-Ki-67 monoclonal antibody (1:100; M7240; Dako), anti-cleaved PARP (Asp214) monoclonal antibody (1:50; clone: D64E10, #5625; Cell Signaling Technology, Danvers, MA, United States), or anti-E-cadherin monoclonal antibody (1:100; M3612; Dako) at 4°C. After washing the sections with PBS, they were incubated with horseradish peroxidase-tagged secondary antibodies (REAL EnVision^TM^ Detection System, Dako) at room temperature for 1 h. Thereafter, the sections were treated with the peroxidase substrate, 3,3′-diaminobenzidine (K5007; Dako). Nuclei were stained with hematoxylin.

### Viable Cell Count and Colony-Forming Assay

To analyze the proliferative potential of the cells in the sheets, viable cells were counted and a colony-forming assay was performed as described in our previous article (Morino et al., 2018). A total of 2,000 live cells from cell sheets were seeded on mitomycin-treated 3T3 cells in KCM containing 1 μM Y-27632. The medium was refreshed on days 5 and 10. After 12 days, the cells were fixed using 4% paraformaldehyde, and stained with crystal violet solution (Merck, Darmstadt, Germany).

### *In vitro* Grafting Assay

An *in vitro* grafting assay was performed as previously reported (Kasai et al., 2017, 2020). Briefly, a harvested nasal cell sheet was attached onto type I collagen gel in a 60 mm dish, and cell cultivation was continued for 7 days in KCM containing 1 μM Y-27632.

### Statistical Analysis

Statistical analysis was performed using the GraphPad Prism 7.0 software (GraphPad, Inc., La Jolla, CA, United States). The mean values in a two-sample comparison were determined by Student’s *t*-test and expressed as *P*-values. The comparison of mean values of multiple sample groups was done by Bonferroni and Tukey–Kramer multiple comparison tests after one-way analysis of variance. *P*< 0.05 were considered significant.

## Results

### Evaluation of Proliferative Ability of Nasal Tissue After Preservation

**FIGURE 1:**
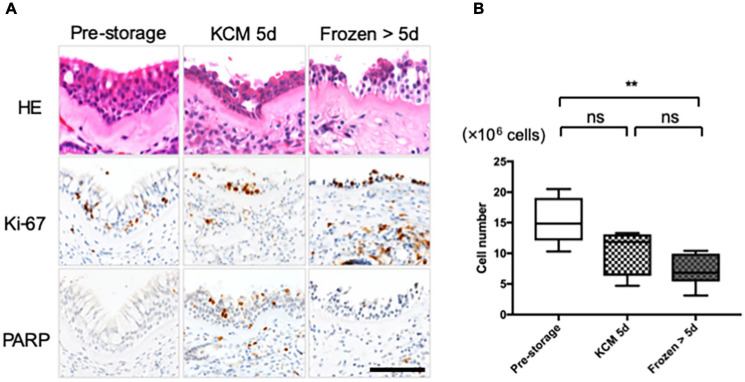
Nasal cells expanded from tissue refrigerated for 5 days and frozen–thawed tissue. **(A)** Hematoxylin and eosin (HE) staining and immunohistological evaluation of the expression of Ki-67 and PARP in pre- and post-storage nasal tissue. **(B)** Cell number was determined for cells cultured from each explant in eight dishes under each condition. Scale bar = 100 μm. **P < 0.01; ns, not significant.

Results of HE staining show the presence of cilia, goblet, and basal epithelial cells in the pre-storage tissue, while some upper layer cells were peeled off from the refrigerated and cryopreserved tissue samples (Figure 1A). To detect the proliferation activity of the cells in fresh tissue stored at 4°C for 5 days (refrigerated tissue) and at −80°C for 19–70 days (cryopreserved tissue), the expression of proliferation marker, Ki-67, and apoptosis marker, poly (ADP-ribose) polymerase (PARP), was examined by immunohistological analysis. Although some upper layer cells were desquamated from the refrigerated tissue, the expression of Ki-67 was observed in all the specimens. PARP was expressed in the refrigerated tissue, suggesting the initiation of apoptosis. For long-term preservation, we also investigated applicability of the cryopreservation method. Although PARP was expressed in cryopreserved tissue, Ki-67 was also expressed. These results indicate that the cell proliferation ability was retained in all tissues.

In the explant culture, cellular outgrowths were observed irrespective of whether preservation was performed. After 13 days of culture, 15.3 ± 3.4 × 10^6^ epithelial cells were collected from fresh tissue samples (Figure 1B, *n* = 6). The difference in the cell number was not significant compared with that obtained from refrigerated tissue samples (10.4 ± 3.4 × 10^6^ cells, *n* = 4; *P* > 0.05; Figure 1B). The cell number in the case of cryopreserved tissue samples was significantly lower than that in the case of fresh tissue (7.1 ± 2.4 × 10^6^ cells, *n* = 6; *P* < 0.01; Figure 1B) but was sufficient for fabricating more than 10 cell sheets.

### Preservation and Its Limits for the Fabrication of Nasal Mucosal Cell Sheets

**FIGURE 2:**
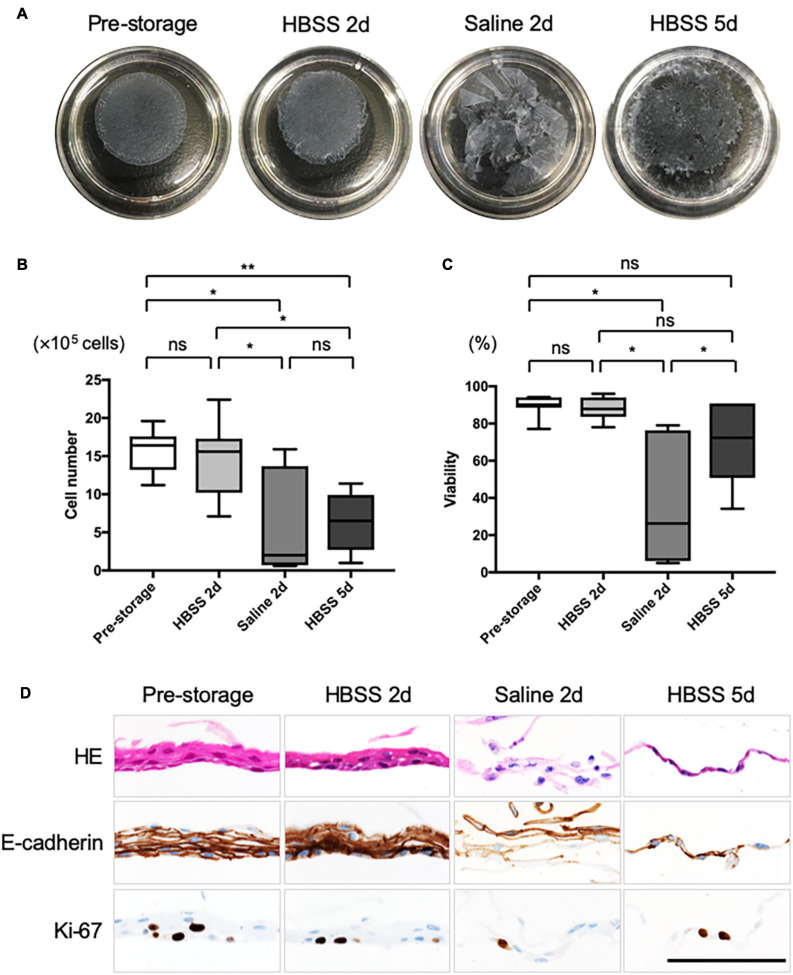
Quality evaluation of post-storage cell sheet. **(A)** Representative images of the cell sheet before and after preservation. **(B)** Cell number was determined from a harvested cell sheet derived from nasal mucosal tissues (*n* = 5) under each condition. **(C)** Cell viability was determined from a harvested cell sheet obtained from nasal mucosal tissue (*n* = 5) under each condition. **(D)** Hematoxylin and eosin (HE) staining and immunohistological evaluation of the expression of E-cadherin and Ki-67 in pre- and post-storage nasal tissues. Scale bar = 100 μm. *P < 0.05; **P < 0.01; ns, not significant.

Feeder supported nasal epithelial cells, derived from the pre-storage tissue samples, formed cell sheets with no defects, contained over 1 million cells (15.8 ± 0.2 × 10^5^ cells, *n* = 7; Figures 2A,B), and exhibited high viability (89.5 ± 2.0%, *n* = 5; Figure 2C). After the cell sheet was preserved in HBSS for 2 days, no significant difference in the cell number (14.6 ± 1.8 × 10^5^ cells, *n* = 5, *P* > 0.05; Figure 2B) or cell viability (87.9 ± 3.5%, *n* = 5, *P* > 0.05; Figure 2C) of the pre-storage cell sheet was observed. In contrast, the shape of cell sheets could not be maintained for 2 days in saline (Figure 2A). In addition, the cell number (6.0 ± 0.6 × 10^5^, *n* = 7, *P* < 0.05; Figure 2B) and cell viability (38.0 ± 9.2%, *n* = 5, *P* < 0.05; Figure 2C) upon storage in saline were significantly lower than the values during pre-storage. After preserving the cell sheet for 5 days in HBSS, it was impossible or difficult to detach an intact cell sheet (Figure 2A). Cell viability in the post-storage cell sheet in HBSS for 5 days tended to be lower than that during pre-storage (70.7 ± 22.3%, *n* = 7, *P* > 0.05; Figure 2C). Although the difference was not significant, the cell numbers in the cell sheet stored in HBSS for 5 days were significantly lower than those during pre-storage (6.3 ± 1.3 × 10^5^ cells, *n* = 7, *P* < 0.05; Figure 2B). These results indicate that HBSS is suitable for preserving nasal mucosal cell sheets for 2 days.

As observed for cells derived from either refrigerated or cryopreserved tissue samples, the cell sheet could be detached in an intact condition (Supplementary Figures 1A,D). Although the cell number and viability were not significantly changed in pre- and post-storage samples (Supplementary Figures 1B,C,E,F), it was not possible to detach the cell sheet without any defect after 2 days in saline or after 5 days in HBSS. These results are similar, as the cell sheets were derived from fresh tissues.

### Histological Evaluation of Nasal Mucosal Cell Sheets

We performed HE staining and immunohistological analysis of cell sheets (Figure 2D). HE staining showed that pre-storage cell sheets were composed of approximately 2–5 layers of squamous epithelial cells; however, no cilia or secreting cells were observed. Moreover, the structure of cell sheet was maintained for 2 days in HBSS even if the cell sheets cells were derived from refrigerated or cryopreserved tissue samples (Figure 2D and Supplementary Figures 2A,B). In contrast, the cell sheet could barely be sampled for 2 days in saline derived from pre-preserved tissue (Figure 2D), it was impossible to sample for 2 days in saline derived from post-preserved tissues^—^there were no data in Supplementary Figures 2A,B. E-cadherin was expressed in almost all cells within normal cell sheets and in cell sheets preserved for 2 days in HBSS, whereas its expression was not strong in cell sheets preserved in saline for 2 days. Based on the analysis of expression of Ki-67, cells in the sheets could proliferate for 2 days in HBSS. Ki-67 was expressed in the cell sheets at 5 days after preservation in HBSS. We believe that the upper layer cells, rather than the basal proliferating cells, were peeled off as dead cells. These results support the notion that HBSS can maintain the quality of cell sheets for 2 days.

### Proliferative Ability of Nasal Mucosal Cell Sheets Is Maintained for a Few Days

**FIGURE 3:**
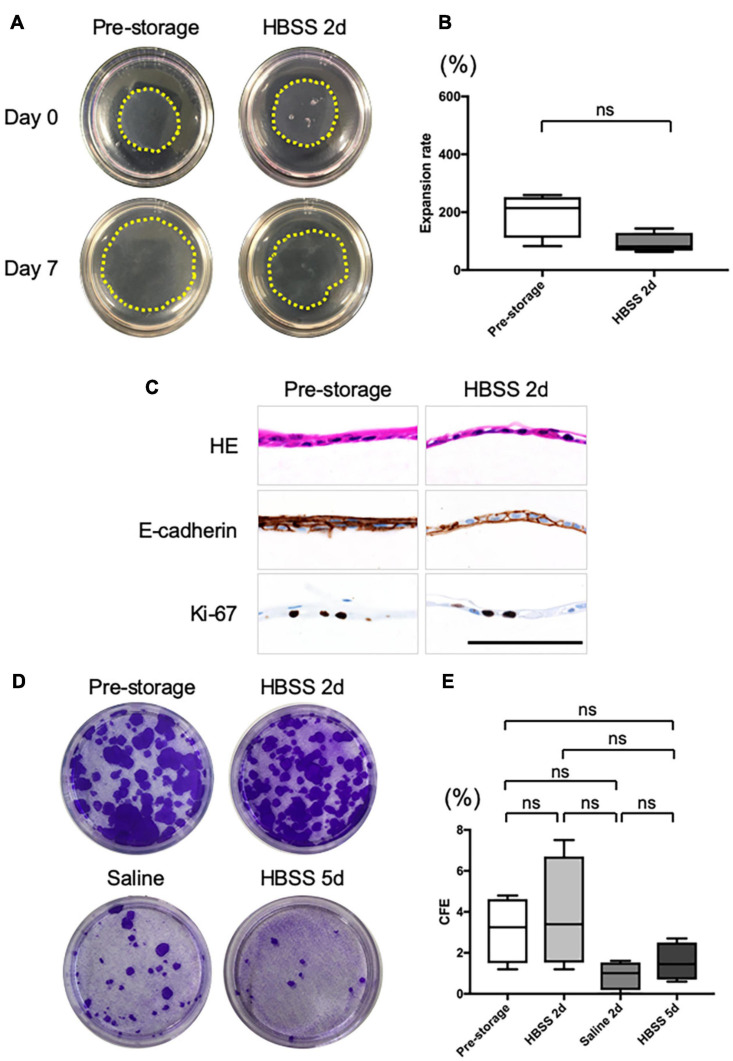
*In vitro* evaluation of the cell sheet after grafting. **(A)** Representative images of pre- and post-storage cell sheets, 0 and 7 days after grafting on type I collagen gels in 60 mm dishes. The yellow dotted line shows the edge of grafted cell sheets. **(B)** Expansion rate of cell sheet size from day 0 to day 7 after grafting (*n* = 6). **(C)** Hematoxylin and eosin (HE) staining and immunohistological evaluation of the expression of E-cadherin and Ki-67 in cell sheet 7 days after grafting on collagen gel. **(D)** Representative images of colony-forming assays for pre- and post-storage cell sheets under each condition. **(E)** Colony-forming efficiency (CFE). Values are expressed as the mean ± SEM (*n* = 6) values. ns, not significant.

To assess the proliferation potential of cell sheets before and after preservation, we performed an *in vitro* cell sheet grafting assay. Because samples of cell sheets preserved for 2 days in saline or for 5 days in HBSS could not be detached intact, they were not amenable to *in vitro* assays. All samples of normal cell sheet and cell sheets preserved for 2 days in HBSS successfully adhered and migrated to the collagen gel (Figure 3A). The expansion rate at 7 days after grafting was not significantly different from that of pre-storage cell sheets (193 ± 67%, *n* = 4) and of cell sheets stored in HBSS (92 ± 31%, *n* = 4, *P* > 0.05; Figure 3B). Moreover, the expansion rate of the cell sheet derived from refrigerated tissue samples was 342 ± 33% (*n* = 4) and for the cell sheet stored in HBSS was 405 ± 82% (*n* = 4, *P* > 0.05; Supplementary Figures 2A,B); the expansion rate of the cell sheet derived from frozen tissue samples at 7 days after grafting was 303 ± 27% (*n* = 4) and for the cell sheet stored in HBSS was 314 ± 64% (*n* = 4, *P* > 0.05; Supplementary Figures 2C,D). We performed HE staining and immunohistological analysis of cell sheets derived from pre-storage (Figure 3C), refrigerated (Supplementary Figure 3E), and cryopreserved (Supplementary Figure 3F) tissue samples at 7 days after grafting. HE staining showed that the pre-storage cell sheets and those stored in HBSS adhered to the collagen gel. Immunohistological analysis showed that E-cadherin connected each cell, enabling the collective migration as observed in epithelial wound healing. Moreover, Ki-67 was still expressed in the grafted cell sheets. These findings indicate that the proliferation ability and wound healing potential of nasal mucosal cell sheets stored in HBSS was retained for 2 days.

Colony-forming assays (Figure 3C) showed that the colony-forming efficiency (CFE) of a normal cell sheet was 3.1 ± 1.4% (*n* = 4; Figure 3D). The CFE score of cell sheets at 2 days after preservation in HBSS showed no significant difference (3.9 ± 2.3%, *n* = 4, *P* > 0.05; Figure 3D). The CFE of the cell sheet at 2 days after preservation in saline was 0.9 ± 0.6% (*n* = 4; Figure 3D) and at 5 days after preservation in HBSS was 1.5 ± 0.8% (*n* = 4; Figure 3D); the value tended to be lower than that at pre-storage, although the difference was not significant. In addition, the CFE scores of cell sheets derived from tissue samples refrigerated for 5 days and from frozen tissue samples were similar to those obtained for cell sheets derived from fresh tissue samples (Supplementary Figure 3).

## Discussion

**FIGURE 4:**
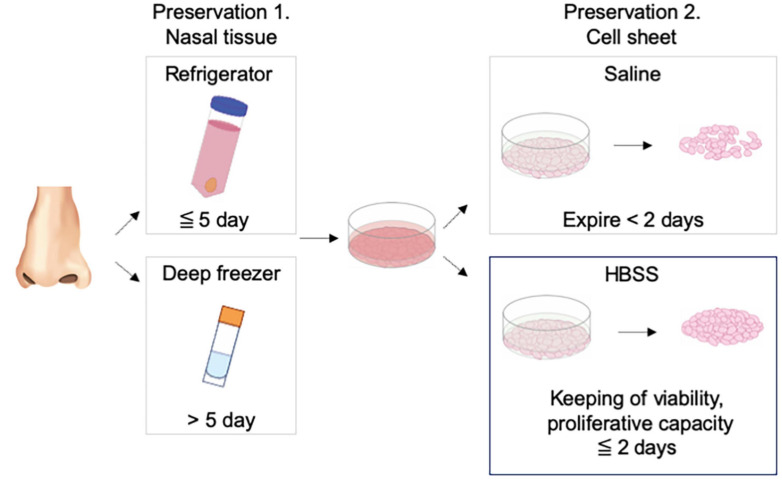
Schematic diagram for the study. Nasal mucosal cell sheet could be fabricated from nasal mucosal tissue stored in a refrigerator or deep freezer and stored in Hank’s balanced salt solution for 2 days in a ready-to-use condition.

Tissue preservation has important effects on precise analysis, cell culture, and tissue transportation in regenerative medicine (Takagi et al., 2015; Mizuno et al., 2017). We found that nasal cells cultured from refrigerated and frozen–thawed tissues formed nasal mucosal cell sheets. In the refrigeration method, KCM maintained the cell proliferation ability for 5 days, which is sufficient for short-term preservation and domestic transportation (Figure 4).

Two-stage operation is an effective technique for treating middle ear cholesteatoma and is performed 6–12 months after the first operation (Ho and Kveton, 2003; Kojima et al., 2006). If the nasal mucosal tissue can be preserved longitudinally, it would be useable for the second surgery. According to Shimazu et al., Stem-Cellbanker^®^ is the best medium for cryopreservation of umbilical cord tissue (Shimazu et al., 2015). Based on their study, we used Stem-Cellbanker^®^ to cryopreserve nasal mucosal tissue and succeeded in fabricating cell sheets (Supplementary Figures 1D–F), although the cell number was lower than that in cells sheets derived from fresh tissue on day 13 (Figure 1B). Therefore, cryopreservation may be used for the two-stage operation. Moreover, because part of the nasal mucosal tissue is discarded during nasal surgery, the cryopreservation technique may be useful for further basic research.

HBSS used for preservation of cell sheets contains inorganic ions (i.e., calcium ions) and glucose, which contribute to calcium-dependent adhesion via E-cadherin (Takeichi, 1977). The cell number and viability of cells in the cell sheet were maintained for 2 days (Figures 3A–C). Because calcium ions are not present in saline, calcium dependent cell–cell adhesion was not retained in cell sheets preserved in saline (Figure 2D). In other studies, a retinal pigment cell sheet was preserved for 5 h (Hori et al., 2019) and an oral mucosal cell sheet was preserved for 12 h (Oie et al., 2014). Therefore, cell sheet quality can be maintained in HBSS for 2 days, which is long enough for stability studies and for transport throughout Japan. This time period is longer than that reported in our previous study (3 h) (Kasai et al., 2019).

It is important that a cell sheet maintains its proliferative potential in a manner similar to that observed for other regenerative medicines (Rama et al., 2010; Butler et al., 2016; Islam et al., 2017). Epithelial cell sheet is known to be applicable for wound healing (Gallico et al., 1984; Nishida et al., 2004). The wound healing-like behavior of cell sheet has been observed using *in vitro* grafting assay (Kasai et al., 2017). Similarly, cell sheet preserved for 2 days in HBSS expressed Ki-67 after grafting and showed wound healing-like behavior, indicating its proliferation on the collagen gel (Figure 3). Thus, our results show the wound healing potential of cell sheets preserved for 2 days in HBSS.

There are some limitations to this study. First, we preserved the cell sheets only at room temperature. Hori et al. preserved retinal pigment epithelium cell sheets at 37°C (Hori et al., 2019), and Kawazoe et al. preserved skin grafts at 4°C (Kawazoe et al., 2008). Therefore, optimization of temperature may be one of the key factors for long-term preservation of cell sheets. Second, considering mass production, we used foreign substances, including gamma-irradiated fetal bovine serum and X-ray-irradiated 3T3-J2 cells. Although these agents have been used for the development of JACE^®^ as a regenerative medicine product, which has an excellent safety record, the residues in the final product must be analyzed. Third, we did not precisely characterize the components of various cell types in the cell sheets before and after grafting. Because nasal mucosal tissue contains different cell types, including basal epithelial cells, goblet cells, and ciliated cells, precise mRNA and protein assays are important to understand the safety and effectiveness of the nasal mucosal cell sheet. Finally, although the wound healing potential was evaluated *in vitro*, an assessment of the effect of nasal mucosal cell sheet after *in vivo* grafting is still a challenging issue for this regenerative medicine. Further studies are needed to address these limitations.

## Conclusion

Nasal tissue cells maintained their proliferative ability in a cell sheet when stored in a refrigerator for 5 days or in a deep freezer for more than 5 days. Nasal mucosal cell sheets can retain their wound healing potential for 2 days in HBSS, which can be used as a ready-to-use preservative. Our findings may facilitate the fabrication of stable cell grafts for use in regenerative medicine.

## Data Availability Statement

The original contributions presented in the study are included in the article/Supplementary Material, further inquiries can be directed to the corresponding author/s.

## Ethics Statement

This study was approved by the Institutional Review Board of the Jikei University, Tokyo, Japan (approval number: 26-359). Informed consent was obtained from all volunteers. The patients/participants provided their written informed consent to participate in this study.

## Author Contributions

YK, TM, KY, and HK contributed to conception and design of the study. YK, ID, and EM performed the experiments. YK wrote the first draft of the manuscript. TM, ID, and KY revised the manuscript. HK supervised this project. All authors approved the original and revised versions of the manuscript.

## Conflict of Interest

The authors declare that the research was conducted in the absence of any commercial or financial relationships that could be construed as a potential conflict of interest.

## Abbreviations

CFE: colony-forming efficiency
HBSS: Hank’s balanced salt solution
HE staining: hematoxylin-eosin staining
KCM: keratinocyte culture medium
PARP: poly (ADP-ribose) polymerase.
