# Supplementary material for: Temporary Storage of the Human Nasal Tissue and Cell Sheet for Wound Repair
Source: Front Bioeng Biotechnol. 2021 Jul 21;9:687946. doi: 10.3389/fbioe.2021.687946 (PMC8334733; doi:10.3389/fbioe.2021.687946)
Supplement: Supplementary file 1 [file Data_Sheet_1.PDF]

# *Supplementary Material*

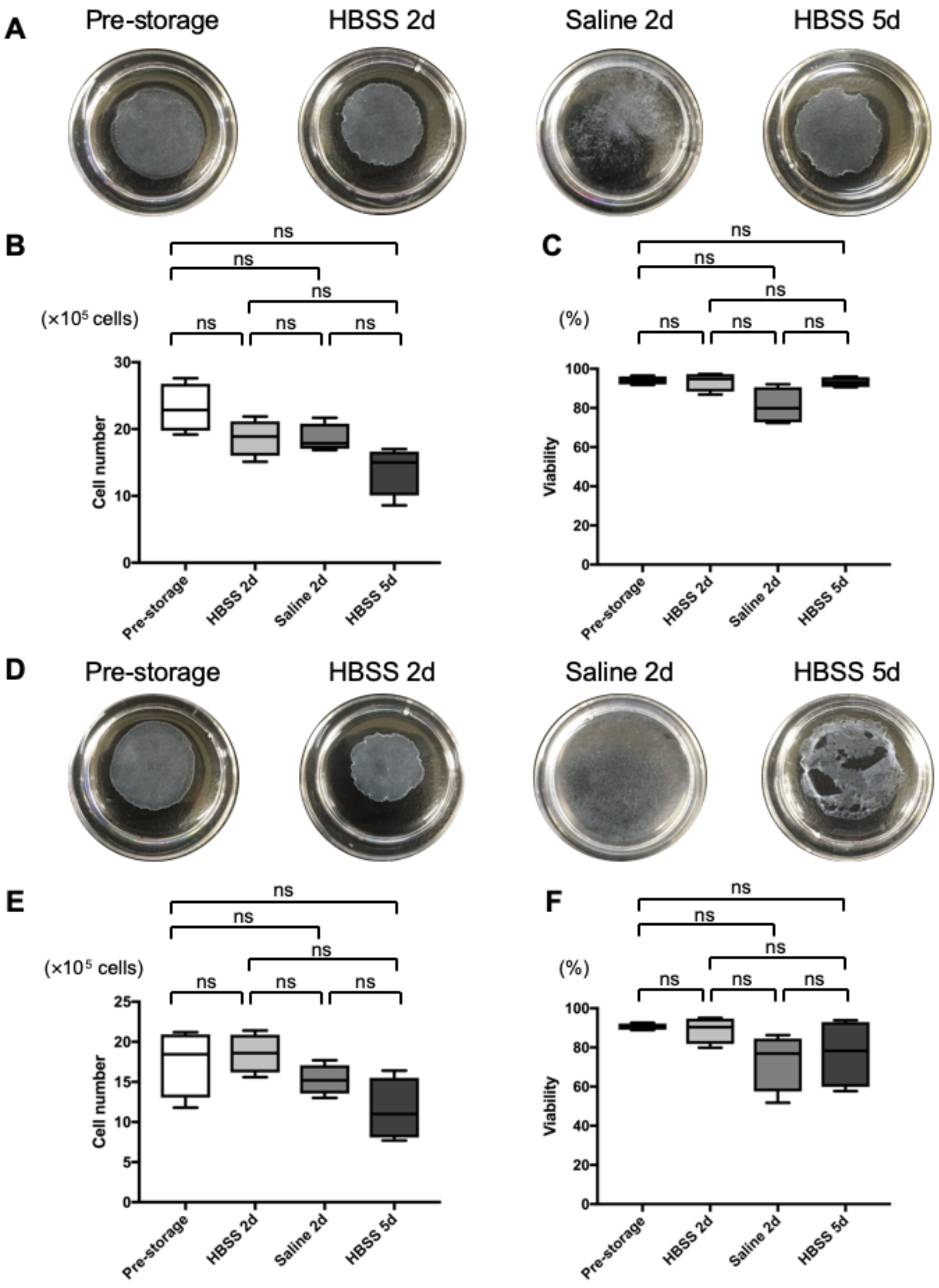


**Supplementary Figure 1.** Quality evaluation of preserved cell sheet obtained from preserved tissue. This Figure is related with Figure 2. (A) Representative images of cell sheets before and after the preservation of refrigerated nasal mucosal tissues for 5 days in KCM. (B) Under each condition, the cell number of cell sheet derived from nasal mucosal tissues refrigerated for 5 days in KCM (n = 4). (C) Under each condition, cell viability of cell sheet derived from refrigerated tissue (n = 4). (D) Representative images of a cell sheet before and after preservation; the cell sheet was obtained from frozen-thawed nasal mucosal tissue. (E) Under each condition, the cell number of a cell sheet derived

from frozen-thawed nasal mucosal tissues (n = 4). (F) Under each condition, the cell viability of a cell sheet derived from frozen-thawed nasal mucosal tissue (n = 4).

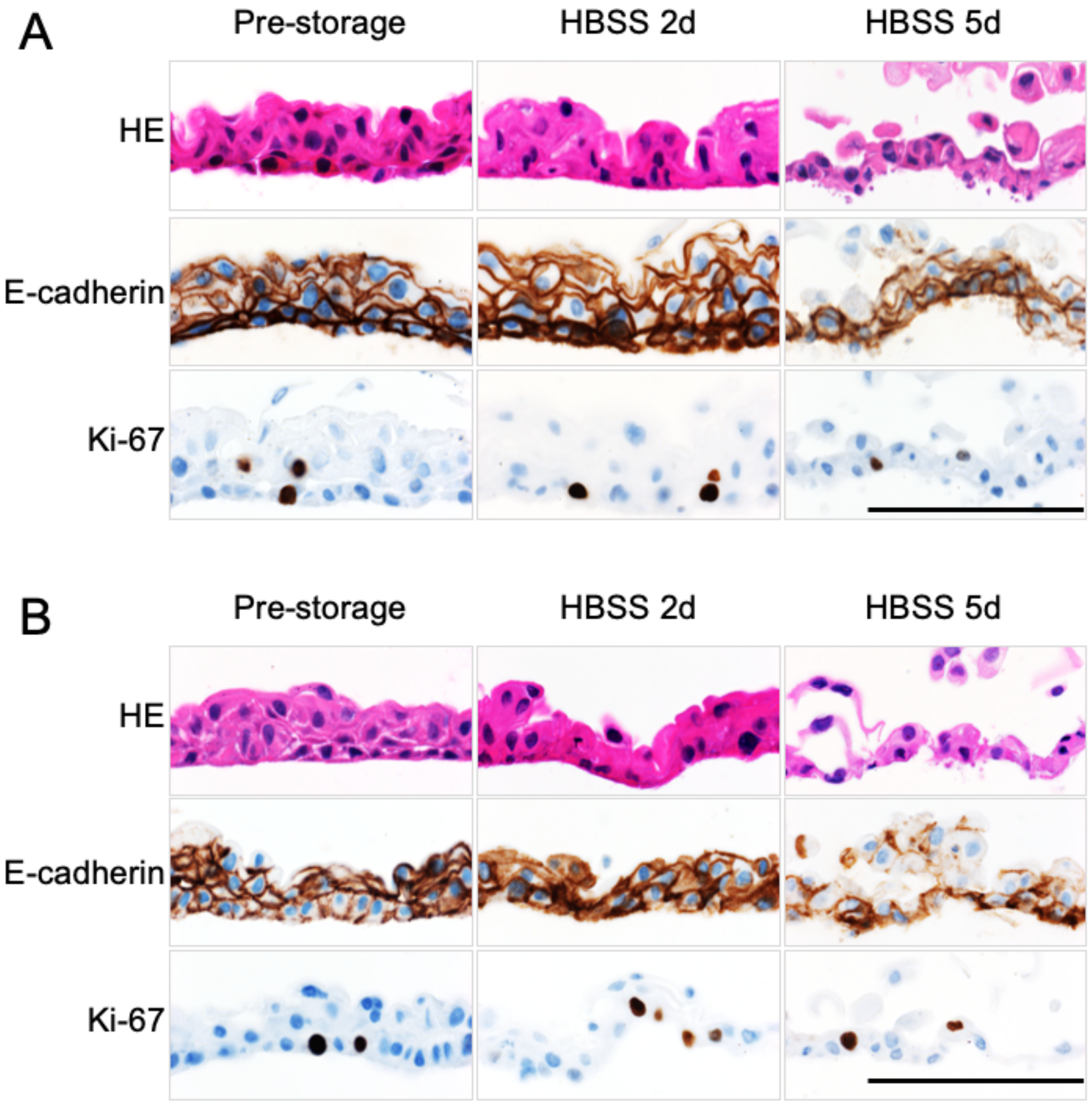


**Supplementary Figure 2.** Histological analysis of cell sheet pre- or post-storage in HBSS for 2 and 5 days, derived from post storage tissue. This Figure is related with Figure 2D. (A) Nasal mucosal cell sheet derived from refrigerated tissue. (B) Nasal mucosal cell sheet derived from frozen-thawed tissue. The top of each panel is labeled with the cell sheet pre- or post-storage in HBSS for 2 and 5 days. The left of each panel is labeled with the protein of interest. Scale bar = 100 µm.

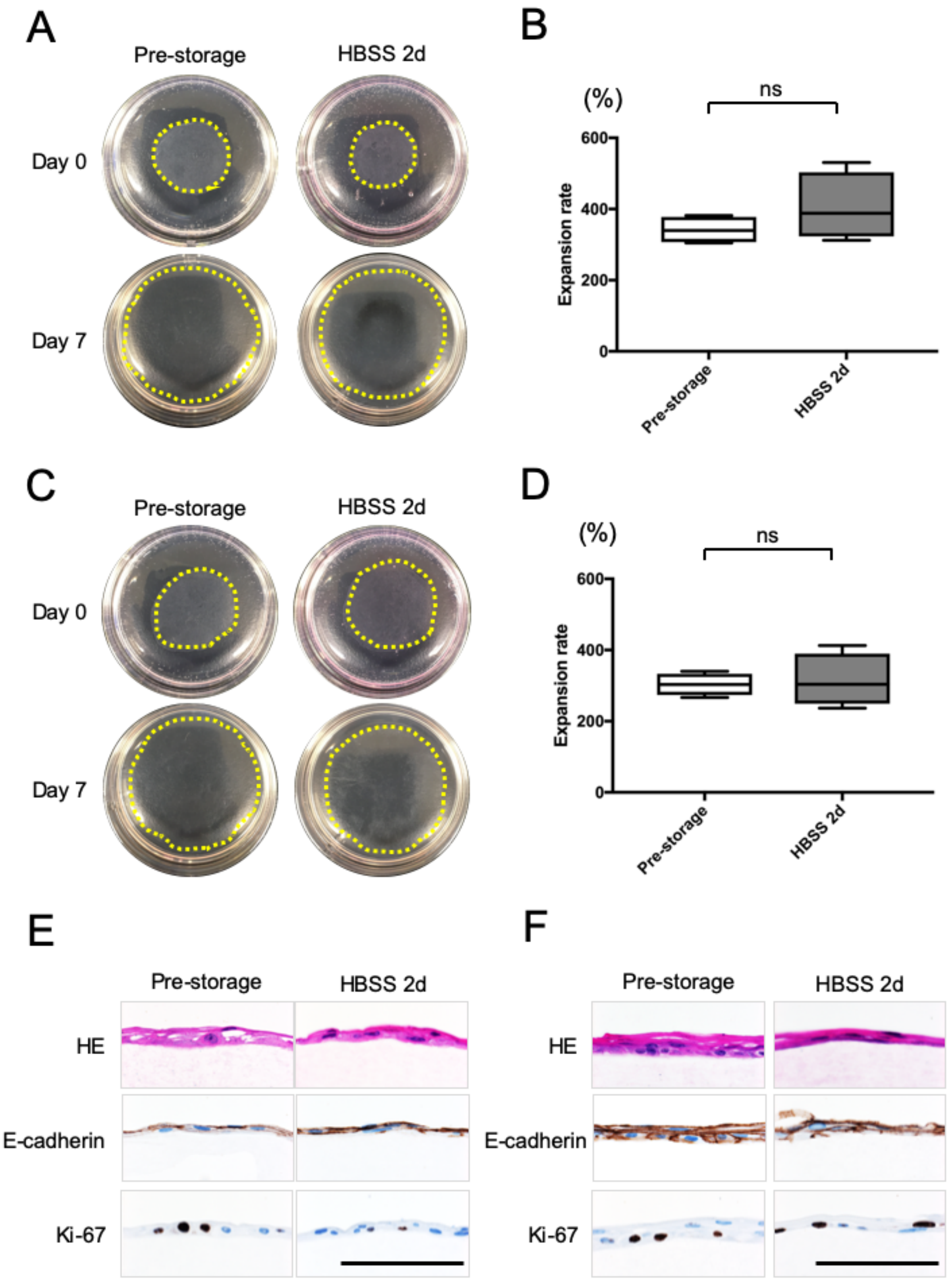


**Supplementary Figure 3.** In vitro evaluation for post-grafting cell sheet. This Figure is related with Figure 3A, B, C. (A) Representative images of cell sheets 0 and 7 days after grafting which derived

from refrigerated tissue on type I collagen gels. (B) Expansion rate of cell sheet area derived from refrigerated tissue from day 0 to 7 after grafting (n = 4). (C) Representative images of cell sheets 0 and 7 days after grafting which derived from frozen-thawed tissue on type I collagen gels. (D) Expansion rate of cell sheet area derived from frozen-thawed tissue from day 0 to 7 after grafting (n = 4). The yellow dotted line shows the edge of grafted cell sheets. (E) Histological analysis of cell sheet derived from refrigerated tissue 7 days after grafting on collagen gel. (F) Histological analysis of cell sheet derived from frozen-thawed tissue 7 days after grafting on collagen gel. The left of each panel is labeled with the protein of interest. Scale bar = 100 μm.

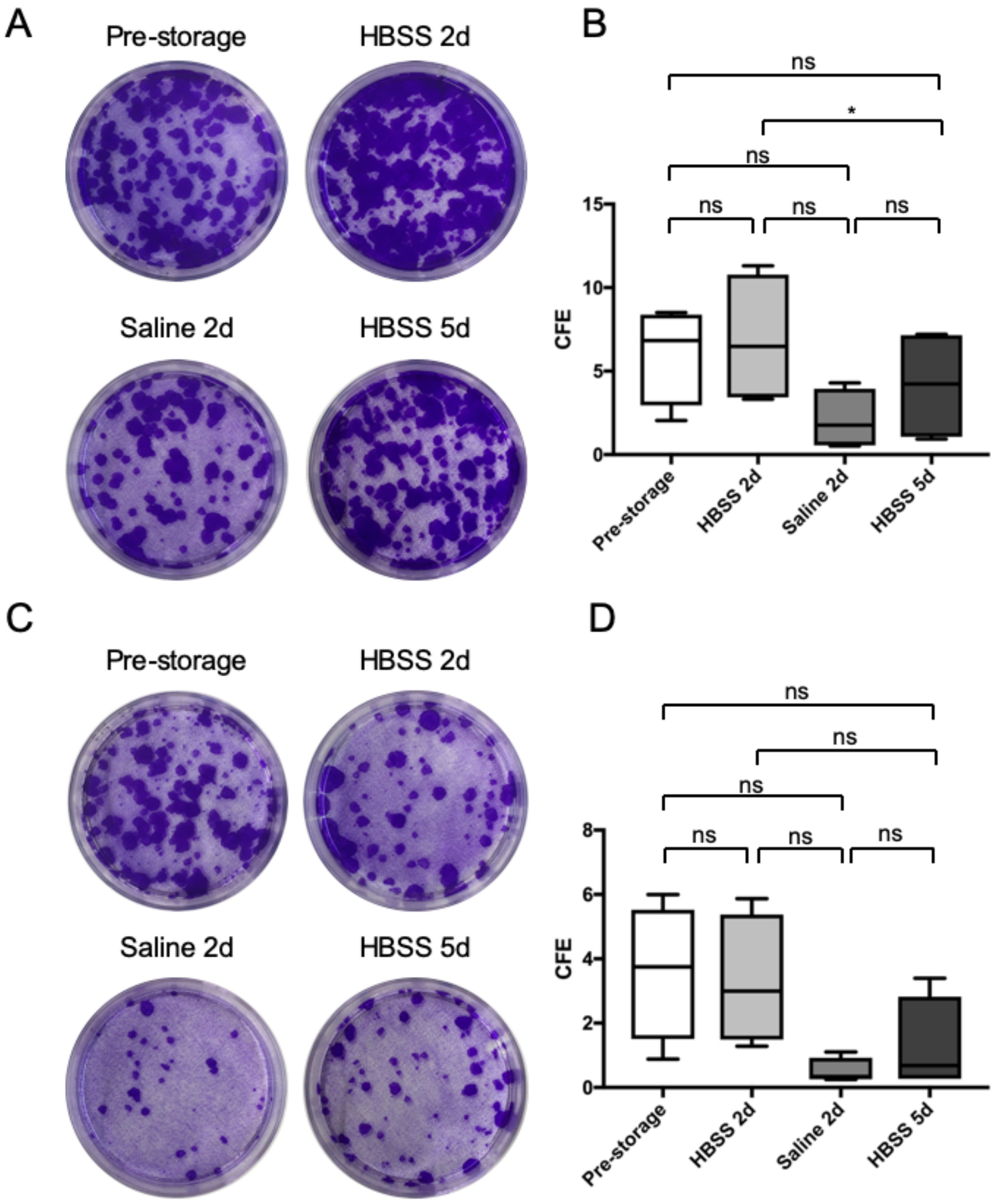


**Supplementary Figure 4.** (A) Representative images of colony-forming assays for pre- and post-preserved cell sheets derived from refrigerated tissue under each condition are shown. (B) Colony forming efficiency (CFE) of cell sheet derived from refrigerated tissue. Values are expressed as the mean ± SEM (n = 4) values. (C) Representative images of colony-forming assays for pre- and post-preserved cell sheets derived from frozen-thawed tissue under each condition are shown. (D) Colony forming efficiency (CFE) of cell sheet derived from frozen-thawed tissue. Values are expressed as the mean ± SEM (n = 4). ns, not significant.
